# Shewanella putrefaciens: A Critically Emerging Pathogen of Ventilator-Associated Pneumonia

**DOI:** 10.7759/cureus.38858

**Published:** 2023-05-10

**Authors:** Kevin Huynh, Yazan Abdeen

**Affiliations:** 1 Medical School, College of Osteopathic Medicine, Sam Houston State University, Houston, USA; 2 Pulmonary and Critical Care Medicine, Pulmonary and Sleep Physicians of Houston, Webster, USA

**Keywords:** emerging pathogens, rare pathogens, pulmonary critical care, hospital-acquired pneumonia, shewanella putrafaciens, ventilator-associated pneumonia

## Abstract

*Shewanella putrefaciens* is a critically emerging cause of ventilator-associated pneumonia (VAP). *S. putrefaciens* ​​​​​is an oxidase-positive, nonfermenting, hydrogen-sulfide-producing, gram-negative bacillus. Worldwide, there have been six reported cases of ​​​​​​pneumonia and two reported cases of VAP, both caused by *S. putrefaciens*​​​​​. In this study, we discuss the case of a 59-year-old male who presented to the emergency department with altered mental status and acute respiratory distress. He was intubated for airway protection. Eight days following intubation, the patient developed symptoms consistent with VAP, and bronchoalveolar lavage (BAL) revealed *S. putrefaciens*​​​, an emerging nosocomial and opportunistic pathogen, as the causative agent. The patient was treated with cefepime with the resolution of symptoms.

## Introduction

Ventilator-associated pneumonia (VAP) is a common hospital-acquired condition that can lead to significant morbidity and mortality and prolonged hospital stay. It is one of the leading causes of death in ventilated patients [1]. VAP is defined as pneumonia occurring 48 hours after the initiation of intubation [2]. Bacterial causes are generally isolated via bronchoalveolar lavage (BAL) or endotracheal aspirate collection. The most common organisms in VAP include Staphylococcus aureus, Pseudomonas aeruginosa, *Streptococcus* spp., and other gram-negative bacilli (*Escherichia coli, Klebsiella pneumoniae, Acinetobacter *spp., and *Enterobacter *spp.) [3]. However, other gram-negative organisms are increasingly reported as important etiologies in VAP; *Shewanella putrefaciens* is a critically emerging cause of VAP. In this case, we describe a 59-year-old male who developed VAP due to *S. putrefaciens*, a gram-negative bacillus that has rarely been reported as a cause of VAP. To our knowledge, this is the third reported case of VAP due to *S. putrefaciens* [4-6] and a total of six cases associated with pneumonia. Of those six cases, three were exposed to water sources, the natural reservoir of this bacterium [4-6,7].

## Case presentation

**Figure 1:**
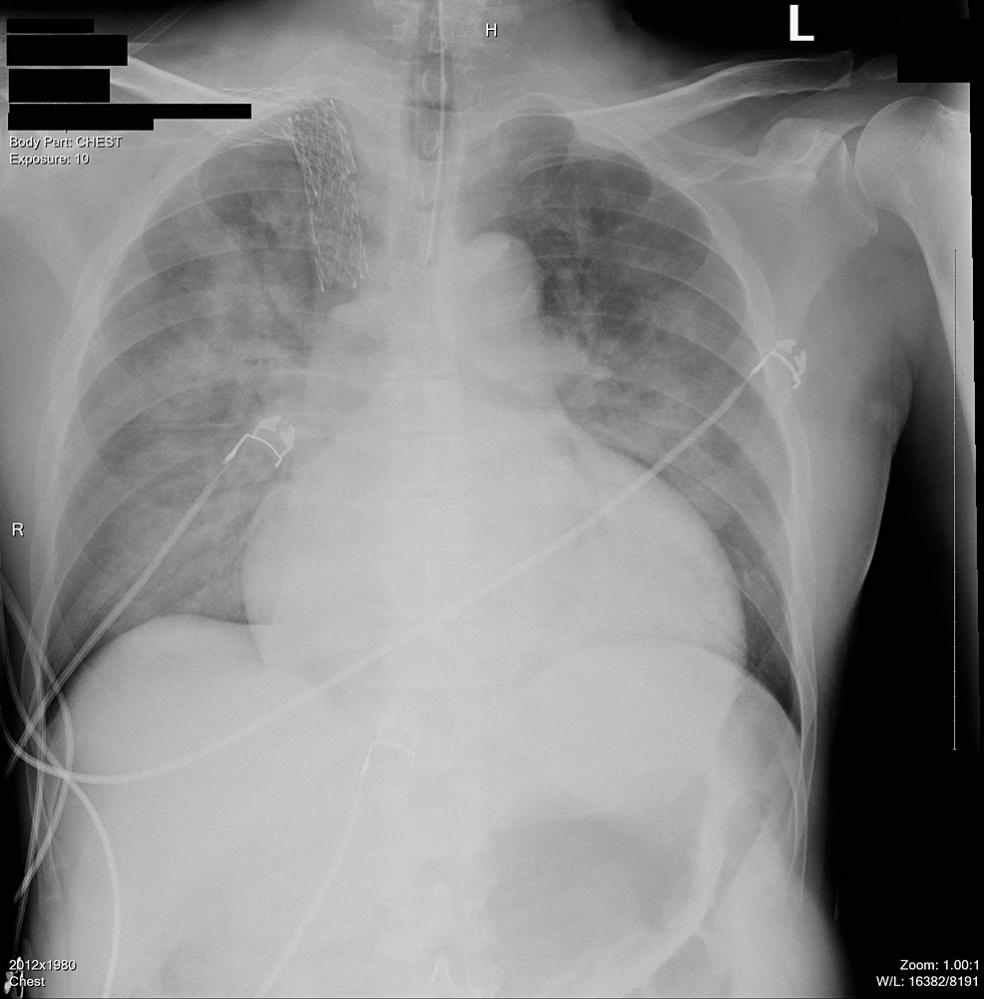
Chest X-ray showing bilateral interstitial, fluffy infiltrates, and ground-glass opacities consistent with pulmonary edema and cardiomegaly.

A 59-year-old male with a history of hypertension, atrial fibrillation, and end-stage renal disease (ESRD) on hemodialysis presented to the emergency department with altered mental status and shortness of breath. Upon presentation to the emergency department, he was found obtunded and in respiratory distress. Physical exam revealed bilateral rales on chest auscultation and jugular venous distension with bilateral lower limb edema. He was subsequently intubated and admitted to the intensive care unit. His initial vital signs showed evidence of hypertensive emergency with a blood pressure of 221/117 mmHg, pulse rate of 99 beats per minute (bpm), and temperature (*T*) of 36.7 °C. The patient had missed his last session of hemodialysis (HD), and the labs were remarkable for hyperkalemia, troponinemia, and elevated ammonia. Initial workup showed evidence of pulmonary edema on chest X-ray (CXR) with no leukocytosis (Figure 1). The CT head was unremarkable.

**Figure 2:**
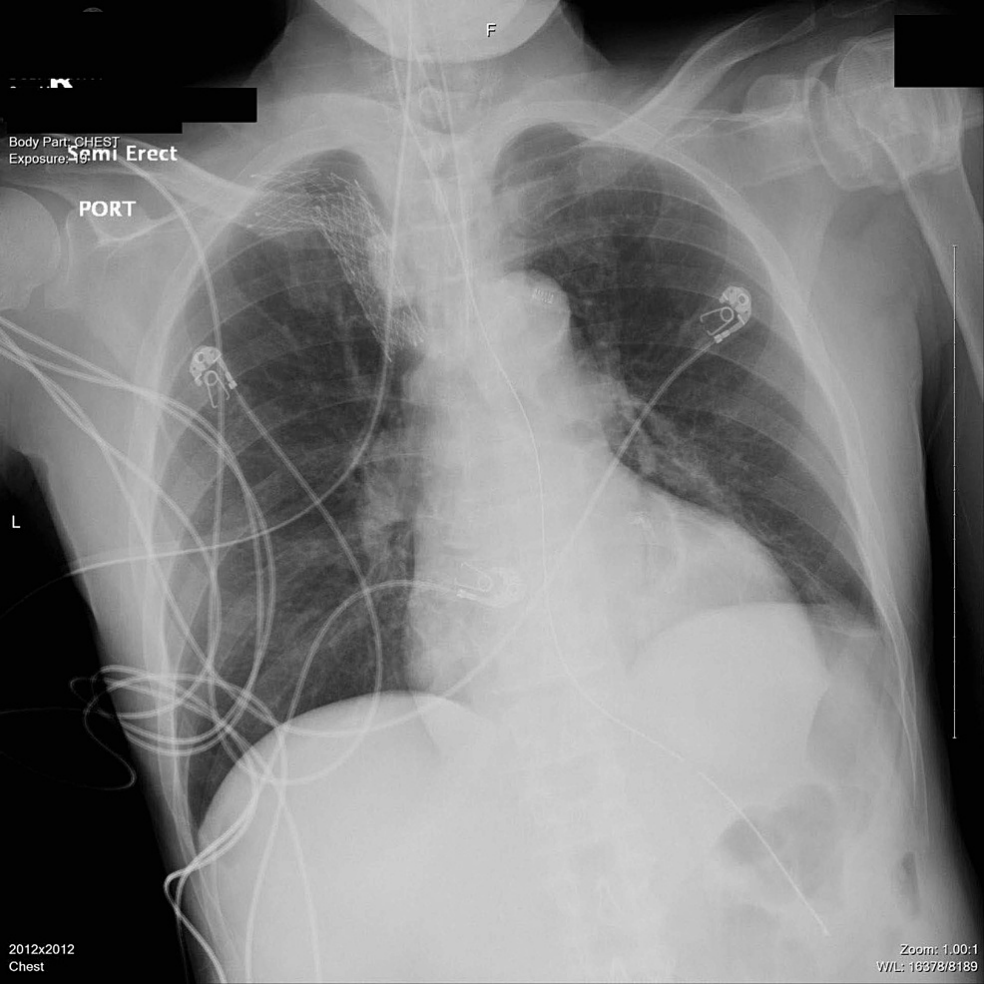
Chest X-ray revealed the resolution of infiltrates compared to the previous X-ray.

Emergent hemodialysis was conducted along with blood pressure control. The patient was treated empirically with intravenous (IV) ceftriaxone for five days, given infiltrates on CXR, and daily HD was conducted for three days with improvement in his volume status and electrolytes. Initially, his respiratory status improved with decreased oxygen requirements, and repeat CXR revealed the resolution of pulmonary edema (Figure 2). Sputum cultures collected via BAL were negative after the course of ceftriaxone. However, he could not be extubated due to poor mental status despite ruling out central brain etiology and optimizing metabolic abnormalities.

**Figure 3:**
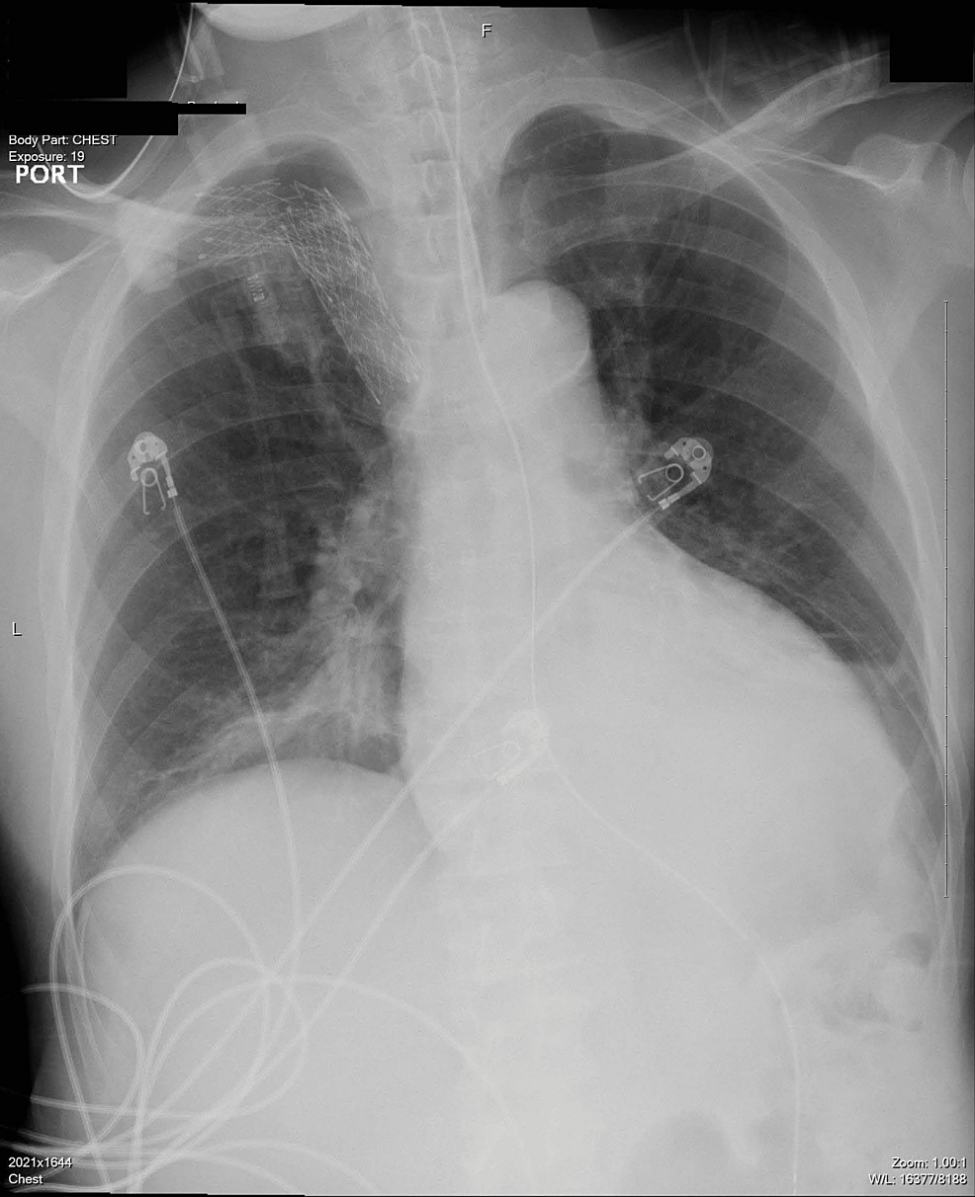
Chest X-ray revealed a new infiltrate in the posterior segment of the right upper lobe.

On day 8 of mechanical ventilation, he developed a fever of 38.5 °C and increased secretions. Workup revealed leukocytosis and a new right upper lobe infiltrate on CXR (Figure 3). He underwent percutaneous tracheostomy and bronchoscopy. On bronchoscopy with lavage of the right upper lobe, he was noted to have thick, yellow-brown secretions with a distinct, malodorous smell. BAL cultures grew *S. putrefaciens.* Culture sensitivities were performed (Table 1), and the patient was started on cefepime and completed a 10-day antibiotic course, with significant improvement in his clinical condition. He was discharged in a stable condition.

**Table 1:** Culture sensitivities from bronchoalveolar lavage. MIC, minimum inhibitory concentration; RX, interpretation; S, sensitive

	Quantitation of *Shewanella putrefaciens*	
	MIC (μg/mL)	RX
Ciprofloxacin	0.5	S
Cefepime	4	S
Gentamicin	<4	S
Meropenem	<1	S
Levofloxacin	<0.5	S
Piperacillin/Tazobactam	<16	S
Tobramycin	<4	S
Trimethoprim/Sulfamethoxazole	<2/38	S

## Discussion

*S. putrefaciens* is an oxidase-positive, nonfermenting, hydrogen-sulfide-producing, gram-negative bacillus [8]. Only *Shewanella algae* and *S. putrefaciens* have been reported to infect humans, with the vast majority being from *S. algae* [9-11]. It is widespread, and generally thrives in marine flora in warm, humid climates and can be found in different types of water, such as marine, seawater, dairy, soil, and even natural gas reserves [8]. It has also been identified in meat and dairy products [9]. Although *S. putrefaciens* is not normally a pathogen to humans, it may occasionally cause skin and soft tissue infections, otitis media, and bacteremia, particularly in patients with underlying medical illnesses such as chronic kidney disease or immunocompromised status [8,12]. Most cases of otitis media were caused by *S. algae* and reported mainly in Denmark [11]. Pathogenesis is thought to be due to mucocutaneous exposure to marine water or consumption of raw seafood [13]. It has been noted as a nonpathogenic contaminant due to colonization of skin flora in several reported cases [12]. There have also been reports of *S. putrefaciens *causing meningitis and osteomyelitis [4,11].

*S. putrefaciens *is an uncommon cause of lower respiratory tract infections, particularly without a clear exposure to marine water or patient risk factors such as immunocompromised status. A few cases reported *S. putrefaciens* isolated in the lower respiratory tract in patients with tuberculosis, patients on peritoneal dialysis, and patients with bacteremia [11]. These reports do not specify colonization versus infection. However, two previous reports have documented *S. putrefaciens* as the causative agent of VAP, and thus, it is crucial to identify an emerging source of VAP [4-5]. Clinical symptoms are very similar to Vibrio infections [9,11]. Previous case reports of VAP caused by *S. putrefaciens* presented as a fever, thick secretions, and acute respiratory distress, manifested by increased ventilator requirements in the patient receiving mechanical ventilation for at least 48 hours. Lung auscultation may reveal crackles, wheezes, or decreased breath sounds, depending on the extent and location of the infection [4-6]. In these reported cases, imaging has been consistent with bacterial pneumonia, with evidence of consolidation in chest radiographs and documented lab abnormalities that were primarily leukocytosis. The organism was isolated via both BAL cultures and endotracheal aspirate cultures. In a report by Tucker et al., treatment for the first documented VAP caused by *S. putrefaciens* was guided by culture sensitivities and was treated with cefepime [4]. Based on the initial case report by Tucker et al., Patel et al. developed the second case of VAP caused by *S. putrefaciens*, which was also treated successfully with cefepime [5]. A case report by Ullah et al. reported VAP and bacteremia due to *S. putrefaciens*, with initial improvement in symptoms and clearance of blood cultures on cefepime. However, the patient's conditions subsequently worsened and comfort measures were initiated; however, they were unable to rule out coinfection with another organism [7]. Regardless, based on a review of the literature, cefepime appears to be effective in treating pneumonia caused by *S. putrefaciens*. Other antibiotics may also be effective based on culture susceptibilities, but cefepime is the most widely reported.

In our case, the patient presented had underlying medical illnesses, including ESRD, which may make him more susceptible to opportunistic infections. Additionally, he was mechanically ventilated for eight days before diagnostic bronchoscopy. The duration of mechanical ventilation may also be a contributing factor. Given the reports of contamination with* S. putrefaciens, *distal quantitative samples obtained by bronchoscopy may be the best avenue for diagnosis and accurate microbiology and pharmacologic testing.

## Conclusions

VAP is a serious and potentially life-threatening condition that can develop during or after a hospital stay, particularly among patients with underlying health conditions. Early recognition of changes in respiratory status, diagnosis with samples obtained by bronchoscopy, and prompt initiation of appropriate antibiotic therapy are essential for successful management. Although rare,* S. putrefaciens* is a gram-negative bacillus known mostly as a marine flora and has been known to cause opportunistic infections in humans, mostly as soft tissue and skin infections. However, in our case, *S. putrefaciens *presented as a lung infection. There have been only two documented cases of VAP caused by *S. putrefaciens*, and physicians should be aware of its emergence as a pathogen for diagnostic and treatment purposes, particularly in patients with underlying chronic kidney disease, immunocompromised status, or exposure to a water source. 
